# Spatially Restricted G Protein-coupled Receptor Activity via Divergent Endocytic Compartments* [Fn FN2]

**DOI:** 10.1074/jbc.M113.526350

**Published:** 2013-12-27

**Authors:** Frederic Jean-Alphonse, Shanna Bowersox, Stanford Chen, Gemma Beard, Manojkumar A. Puthenveedu, Aylin C. Hanyaloglu

**Affiliations:** From the ‡ Institute of Reproductive and Developmental Biology, Department of Surgery and Cancer, Imperial College London, London W12 0NN, United Kingdom and; the § Department of Biological Sciences, Carnegie Mellon University, Pittsburgh, Pennsylvania 15213

**Keywords:** Endocytosis, Endosomes, G Protein-coupled Receptor (GPCR), Receptor Recycling, Signaling, Sorting, GAIP-interacting Protein C Terminus (GIPC)

## Abstract

Postendocytic sorting of G protein-coupled receptors (GPCRs) is driven by their interactions between highly diverse receptor sequence motifs with their interacting proteins, such as postsynaptic density protein (PSD95), *Drosophila* disc large tumor suppressor (Dlg1), zonula occludens-1 protein (zo-1) (PDZ) domain proteins. However, whether these diverse interactions provide an underlying functional specificity, in addition to driving sorting, is unknown. Here we identify GPCRs that recycle via distinct PDZ ligand/PDZ protein pairs that exploit their recycling machinery primarily for targeted endosomal localization and signaling specificity. The luteinizing hormone receptor (LHR) and β2-adrenergic receptor (B2AR), two GPCRs sorted to the regulated recycling pathway, underwent divergent trafficking to distinct endosomal compartments. Unlike B2AR, which traffics to early endosomes (EE), LHR internalizes to distinct pre-early endosomes (pre-EEs) for its recycling. Pre-EE localization required interactions of the LHR C-terminal tail with the PDZ protein GAIP-interacting protein C terminus, inhibiting its traffic to EEs. Rerouting the LHR to EEs, or EE-localized GPCRs to pre-EEs, spatially reprograms MAPK signaling. Furthermore, LHR-mediated activation of MAPK signaling requires internalization and is maintained upon loss of the EE compartment. We propose that combinatorial specificity between GPCR sorting sequences and interacting proteins dictates an unprecedented spatiotemporal control in GPCR signal activity.

## Teaser

**Background:** Following ligand-induced internalization, GPCRs are sorted by diverse receptor motifs and protein interactions.

**Results:** Distinct GPCRs are targeted to a pre-early endosome compartment for their sorting and MAPK signaling.

**Conclusion:** GPCR sorting motifs and their interacting proteins provide specificity in endosomal targeting and receptor signaling.

**Significance:** We describe a system to reprogram GPCR signaling at an unprecedented spatial level.

## Introduction

G protein-coupled receptors (GPCRs)^3^ represent core communicators of extracellular signals within all physiological systems. Although an individual cell can express a number of GPCRs, an archetypal view of GPCR signaling depicts cell surface-localized receptors activating specific heterotrimeric G protein signaling pathways that, in turn, converge on common downstream pathways. How such linear convergent signals are translated into the highly diverse cellular and physiological responses that are controlled by this superfamily of receptors has been a long-standing biological question. This question has driven our more current understanding of the increasing complexity of these receptor signaling systems, including the ability of an individual GPCR to activate multiple G protein and non-G protein pathways, receptor homo- and heterodimerization, and biased or ligand-directed signaling. How this functional pleiotropy in GPCR signaling is translated to specific downstream cellular responses is poorly understood (1–3).

One mechanism that can potentially regulate both signal specificity and diversity for distinct receptor families, including GPCRs, is membrane trafficking. Endocytic membrane trafficking has emerged from a system with limited cellular roles in the uptake of nutrients, receptors, and plasma membrane to one that is deeply integrated with cell signaling. Consequently, an increasing number of clinical conditions have been reported to result from defects in endocytosis or subsequent sorting of internalized cargo (4–7). Several pathways have been described to regulate cargo entry into the cell, with endocytosis via clathrin-coated pits (CCPs) being the most extensively characterized. For many GPCRs, clathrin-mediated endocytosis occurs via the recruitment and binding of β-arrestins to the activated, phosphorylated GPCR, which inhibits interaction with their cognate G proteins and promotes receptor clustering in CCPs (1, 8). Although internalized receptors can then traverse several endosomal compartments, the early endosome (EE) is classically considered to be the first postendocytic compartment from which receptors are actively sorted to distinct cellular fates. These include trafficking to the late endosome/multivesicular body for lysosomal-mediated degradation and recycling to the plasma membrane via either the default/bulk membrane or regulated pathways (9–11). The latter pathway involves a mechanism used by many GPCRs whereby a diverse set of structural determinants in their cytoplasmic domains are essential in targeting to this regulated recycling pathway (11, 12). This diversity in sorting mechanisms raises the possibility that the postendocytic sorting fate could be programmed for an individual receptor at multiple levels. However, addressing this possibility requires our general understanding of sorting, and how different processes within the endocytic system are coordinated, to be mechanistically dissected in more detail.

For GPCRs, endocytosis and postendocytic sorting not only regulates receptor cell surface density but also the signaling profile. A simple but dramatic example is the contrasting G protein signal responses generated between receptors targeted to either lysosomal or recycling pathways, resulting in either permanent G protein signal termination or G protein signal resensitization/recovery, respectively (11). Despite these known general functional roles for postendocytic sorting, a key question that still remains unanswered is whether the high diversity in GPCR sorting sequences and protein interactions provides combinatorial specificity in their function, given that many GPCR signals converge onto common pathways.

In this study, we provide evidence that specificity in signaling of distinct internalized GPCRs targeted to the regulated recycling pathway can be achieved by endosomal targeting of receptors upstream of the classic sorting EE. Mechanistically, this pre-EE localization requires the recruitment of the postsynaptic density protein (PSD95), *Drosophila* disc large tumor suppressor (Dlg1), zonula occludens-1 protein (zo-1) (PDZ) protein GAIP-interacting protein C terminus (GIPC) and restricts certain receptors from entering EEs. Importantly, we demonstrate that this receptor-driven specificity in spatial organization within postendocytic compartments is critical to activate distinct MAPK signaling responses. This study reveals a novel facet in how the endocytic system can spatially organize signaling receptors and suggests combinatorial specificity in analogous protein interactions as a mechanism for bias in signaling across endosomal compartments, which could be reprogrammed to create highly regulated and distinct signaling profiles.

## EXPERIMENTAL PROCEDURES

### 

#### 

##### Reagents

For visualizing receptors, FLAG-tagged receptors were labeled with either M1 (Sigma) conjugated with Alexa Fluor 555 or Alexa Fluor 647 (Invitrogen), as described (13), or rabbit (Sigma) anti-FLAG antibodies. For immunofluorescence studies on fixed cells and/or Western blotting, antibodies to Rab5a (BD Biosciences), Rab5b (Santa Cruz Biotechnology), Rab5c (Sigma), EEA1 (Cell Signaling Technology), GIPC (provided by Moses Chao, New York University School of Medicine), p42/44 MAPK and phospho-p42/44 MAPK (Cell Signaling Technology), and β-arrestin 1/2 (New England Biolabs). Pertussis toxin (Sigma) was used at 200 μg/ml and Dyngo-4a (Abcam Biochemicals) at 30 μm. Luteinizing hormone (LH) and FSH (National Hormone and Peptide Program, Harbor-UCLA Medical Center) were used at 10 nm, arginine-vasopressin (Bachem) at 1 μm, and isoproterenol (Sigma) at 10 μm. All concentrations of ligands used give maximal cAMP responses from dose-response curves published previously (14–17).

##### Constructs and siRNA Oligos

The plasmids GIPC-GFP, HA-B1AR, 2xFYVE-GFP, APPL1-GFP, and FLAG-human LHR and FSHR were provided by Marilyn Farquhar (University of California, San Diego), Laëtitia Comps-Agrar (Institut de Genomique Fonctionelle Montpellier), Fernando Martin-Belmonte (Universidad Autónoma de Madrid), Pietro De Camilli (Yale University School of Medicine), and Ilpo Huhtaniemi (Imperial College London), respectively. FLAG-human B2AR, FLAG-human V2R 362T, and clathrin light chain-DsRed have been described previously (18–20). LHR-683T was constructed by replacing leucine 683 with a stop codon by site-directed mutagenesis (QuikChange, Stratagene). The chimera of V2T with the last 17 residues of the LHR C terminus was constructed by introducing an EcoRV site into the full-length V2R at residue 362 and into the LHR at residue 682 by site-directed mutagenesis. Then, both the LHR and V2R were digested with EcoRV/Xba1 and ligated to form V2T/LHR C17. Knockdown of GIPC using siRNA was achieved by transfection of duplex RNA oligos (Invitrogen) corresponding to GCCTTCGACATGATCAGCCAGCTT.

Control cells were transfected with non-sense duplex RNA oligos (AATTCTCCGAACGTGTCACG). siRNAs against Rab5 isoforms (a, b, and c) were as described previously (21).

##### Cell Culture and Transfection

HEK 293 and HeLa cells (ATCC) were maintained in DMEM or minimum Eagle's medium containing 10% FCS, glutamine (0.3 mg/ml), and penicillin/streptomycin (100 units/ml) at 37 °C in 5% CO_2_. Transient and stable transfections of HEK 293 cells were performed with Lipofectamine 2000 (Invitrogen) or Effectene (Qiagen). HeLa cells were transiently transfected with JetPEI (Polyplus). For transient expression, cells were assayed 48 h post-transfection, except for siRNA transfections and live imaging of clathrin-Ds-Red- and GFP-tagged GIPC, where cells were assayed 24–96 h post-transfection.

##### Flow Cytometry

Flow cytometry was used to quantitate the internalization and recycling of receptors by measuring the levels of cell surface FLAG-tagged receptors as described previously (22). All experiments were conducted at least three times. The percentage of receptor recycling was calculated from the proportion of internalized receptors (as indicated by a decrease of immunoreactive surface receptors with the agonist compared with unstimulated cells) that was recovered at the cell surface.

##### Coimmunoprecipitation

Cells were incubated with 1 mm of DSP (dithiobis(succinimidyl propionate), Thermo Scientific) for 2 h at 4 °C. The cross-linking reaction was terminated by addition of Tris-HCl to a 10 mm final concentration. Cells were washed twice with cold PBS, collected, and homogenized with lysis buffer (0.5% Triton, 50 mm Tris-HCl, 140 mm NaCl, 0.5 mm EDTA, and protease inhibitors mixture) for 30 min. Next, lysates were centrifuged, and the supernatant was incubated overnight with M2-agarose affinity gel (Sigma). Pellets were washed three times with lysis buffer, eluted by Laemmli gel, and separated on a 12% SDS-PAGE gel for Western blot analysis.

##### Cell Signaling Assays

Cells were serum-starved for 18 h prior to agonist stimulation. Following agonist treatment, cells were rapidly washed in cold phosphate-buffered saline solution and harvested with lysis buffer (1% Triton X-100, 50 mm Tris-HCl (pH 7.4), 150 mm NaCl, 0.5 mm EDTA, and a protease inhibitor tablet (Roche)). Cell extracts were separated on a 12% Tris-glycine polyacrylamide gel and transferred to a nitrocellulose membrane blotted with phospho-p42/44 MAPK antibody or p42/44 MAPK as a loading control. Signal densities were quantified with ImageJ (http://rsbweb.nih.gov/ij).

The measurement of whole cell cAMP was performed as described previously (22). Each treatment was performed in triplicate, and experiments were repeated at least three times. All cAMP concentrations were corrected for protein levels.

##### Confocal Imaging of Live Cells

Receptor trafficking in live cells was monitored by “feeding” cells with conjugated anti-FLAG M1-Alexa Fluor (AF) 555 (15 min, 37 °C) in phenol red-free DMEM containing 5% FBS. Cells were imaged using a TCS-SP5 confocal microscope (Leica) with a ×63 1.4 numerical aperture (NA) objective, and cells were maintained at 37 °C and 5% CO_2_ using an environmentally controlled incubation chamber. Leica LAS AF image acquisition software was utilized. All subsequent Lif image files were analyzed in ImageJ or LAS AF Lite (Leica) to measure endosome diameter size, conversion of time-lapse movies to tiff stacks, and channel separation.

##### Immunofluorescent Staining and Imaging

Cells seeded on coverslips coated with poly-d-lysine (Sigma Aldrich) were incubated with mouse or rabbit anti-FLAG for 15 min prior to agonist stimulation. For agonist-treated cells labeled with anti-mouse FLAG antibody, cells were washed three times in PBS/0.04% EDTA to selectively remove FLAG antibody bound to the remaining surface receptors (23). Cells were fixed with 4% paraformaldehyde in PBS, blocked in PBS/2% FCS/1% BSA (pH 7.4) buffer, and then immunostained using antibodies to Rab5a or EEA1. AF-conjugated secondary antibodies (Invitrogen) were used to visualize primary antibodies. Coverslips were mounted on glass slides using Fluoromount-G (Southern Biotech) and imaged with confocal microscopy as described above, except that imaging was performed at 21 °C.

##### Total Internal Reflection Fluorescence (TIRF) Microscopy

Cells were imaged using a Nikon Eclipse Ti automated inverted microscope outfitted with a temperature-, humidity-, and CO_2_-controlled chamber. Images were acquired with an iXon+ 897 electron multiplying charge-coupled device camera with an Andor ALC with solid-state lasers of 488 nm, 561 nm, and 647 nm as light sources. The cells were imaged live at 37 °C in Opti-MEM (Invitrogen) supplemented with 40 mm HEPES and 10% FBS using a ×60 1.45 NA TIRF objective (Nikon). Time-lapse movies were collected as tiff stacks and analyzed in ImageJ. Cells expressing FLAG-LHR were imaged for 2 min before LH treatment and 15 min after agonist addition, capturing an image every 3 s.

##### Statistical Analysis

Statistical significance was determined using paired Student's *t* test. Differences were considered significant at *p* < 0.05.

## RESULTS

### 

#### 

##### GPCRs Targeted to a Regulated Recycling Pathway Are Differentially Organized within the Early Endocytic Pathway

**FIGURE 1.**
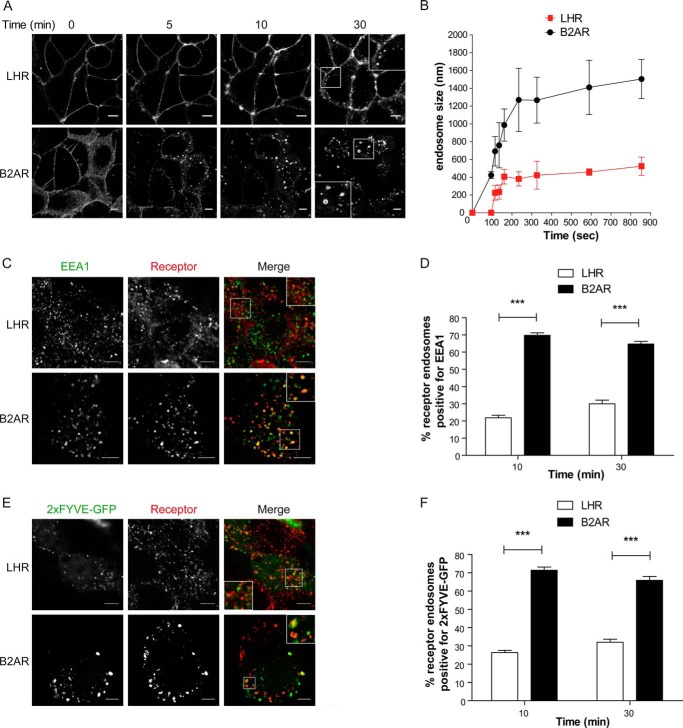
**The LHR and B2AR traffic to distinct compartments in the endocytic pathway.** *A*, HEK 293 cells stably expressing FLAG-tagged LHR or B2AR were labeled with fluorescently tagged anti-FLAG antibodies and imaged live with confocal microscopy before and after agonist treatment. The LHR was stimulated with 10 nm LH, and the B2AR was stimulated with 10 μm isoproterenol. The frames shown were taken from a time-lapse series of supplemental Movies S1 and S2. *B*, the size of the LHR or B2AR containing endosomes was assessed by measuring the diameter of 10 endosomes at each time point stated across three to four movies. Data represent mean ± S.E. *C*, cells expressing either the LHR or B2AR were fed with anti-FLAG antibody and treated with the agonist for 10 min. Cells were fixed, permeabilized, and stained with an anti-EEA1 antibody and imaged with confocal microscopy. The images shown are representative of 16 cells. *D*, numbers of LHRs or B2ARs containing endosomes positive for EEA1 following either 10 or 30 min of agonist stimulation were quantified, and the percentage was calculated. Data are mean + S.E.; *n* = 16 cells, 298 and 295 endosomes respectively for each receptor. *E* and *F*, cells were treated as in *C* and *D* except that cells were transfected with the PI3P marker 2xFYVE-GFP F, and the number of receptor-containing endosomes positive for 2xFYVE-GFP was quantified (*n* = 24 and 22 cells, respectively; LHR and B2AR, ∼980 and 500 endosomes, respectively). The *arrows* represent examples of colocalization. *Scale bars* = 5 μm. ***, *p* < 0.001. See also supplemental Movies S1 and S2.

We compared the trafficking and endosomal targeting of two physiologically relevant Gαs-coupled GPCRs for which membrane trafficking is critical for signaling, the luteinizing hormone receptor (LHR), and the β2-adrenergic receptor (B2AR) (12, 24–26). Upon activation, both the LHR and B2AR are known to bind β-arrestin, undergo clathrin-mediated endocytosis, and are sorted to a sequence-dependent/regulated recycling pathway (12, 18, 20, 27–29). To study the endosomal targeting of these two receptors, we first visualized the dynamics of receptor sorting in live HEK 293 cells stably expressing FLAG-tagged B2AR or LHR using confocal microscopy. Both receptors were observed on the cell surface before addition of the agonist (Fig. 1*A*), whereas after agonist addition, both the B2AR and the LHR internalized and appeared in endosomes within 5 min of agonist stimulation, although this was more evident for the B2AR than the LHR at this time point (Fig. 1*A* and supplemental movies S1 and S2). However, the most striking observation was the physical difference in the size of LHR endosomes compared with B2AR. The larger size of the B2AR endosomes enables visualization of the endosomal lumen and the receptor on the limiting membrane of the endosome, as observed previously in live cells (30, 31). The measurement of endosome size over time revealed that the B2AR enters an endosomal structure of 1200–1400 nm in diameter within 4 min of agonist treatment (Fig. 1*B*). This is a size consistent with prior observations of the recycling Fc receptor trafficking to EEA1-positive EEs in a distinct cell type (32) and, thus, is not a feature of transfection or HEK 293 cells. Although LHR endosome size also increases over time, it is enriched in a smaller endosome population of 400–500 nm where the limiting membrane and lumen are not visible at the resolution level of confocal microscopy. The receptor reaches this endosome population size within 3 min of agonist stimulation (Fig. 1*B*), although there was a delay in the appearance of visible LHR endosomes following 90 s of agonist treatment as compared with the B2AR (Fig. 1*B*).

These observations led us to next investigate the identity of these small LHR endosomes. Either LHR- or B2AR-containing endosomes were covisualized with a classic marker of the EE, early endosome autoantigen 1 (EEA1). The B2AR-containing endosomes colocalized extensively with EEA1 (Fig. 1, *C* and *D*), consistent with previous studies reporting that this receptor traffics to EEs (33, 34). In contrast, the majority of internalized LHR (>70%) were not found in EEA1-positive endosomes following either 10 or 30 min of agonist treatment compared with the B2AR (Fig. 1*D*). The enrichment of endosomes with PI3P is a key feature of the conversion of early endocytic intermediates to EEA1-positive EEs (35). Therefore, we used a lipid biosensor of PI3P to confirm that LHR internalizes to endosomes distinct from the EE. A GFP-tagged 2xFYVE domain of Hrs/Vps27 (36) was transfected into cells expressing either the B2AR or the LHR (Fig. 1, *E* and *F*). Following either 10 or 30 min of agonist stimulation, the internalized B2AR colocalized extensively with the PI3P marker (Fig. 1, *E* and *F*). In contrast, the majority of the LHR endosomes were not positive for 2xFYVE-GFP (Fig. 1, *E* and *F*), suggesting that the LHR endosomal population is primarily PI3P-negative, consistent with the EEA1 localization data.

**FIGURE 2.**
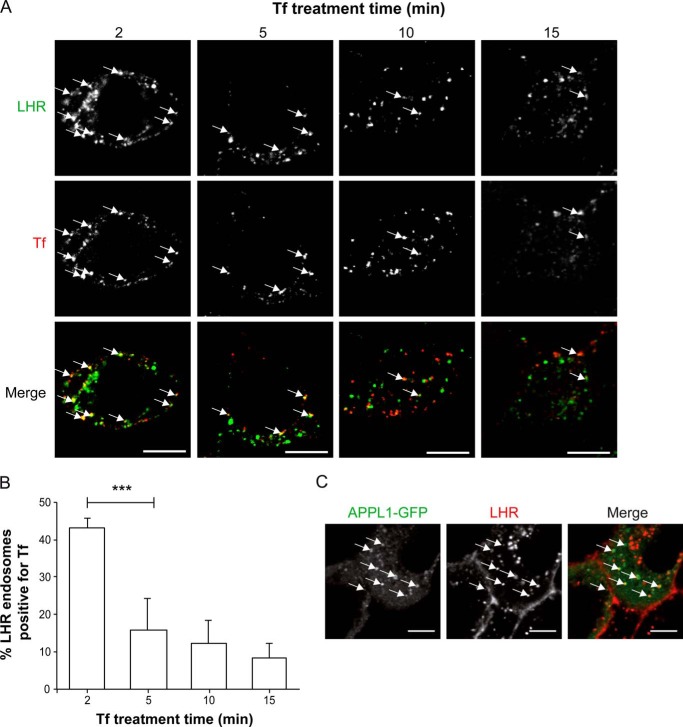
**LHR endosomes are upstream of cargo trafficking to the EE and contain APPL1.** *A*, cells stably expressing FLAG-LHR were fed with anti-FLAG antibody and then treated with the agonist LH (10 nm) for 15 min. AF555-labeled Tf was added to LH-stimulated cells for the final 2, 5, or 10 min of LH treatment. For the 15-min Tf treatment, Tf was added simultaneously with LH. Cells were then washed with PBS/0.04% EDTA (see “Experimental Procedures”) and then fixed and permeabilized for treatment with AF488-labeled secondary antibody. Representative confocal microscopy images from three independent experiments are shown. The *arrows* represent examples of FLAG-LHR and Tf colocalization. *Scale bar* = 5 μm. *B*, numbers of FLAG-LHR endosomes positive for Tf following 2, 5, 10, or 15 min of Tf treatment were quantified, and the percentage was calculated. Data are mean + S.E.; *n* = 8 cells, ∼240 endosomes per condition. ***, *p* < 0.001. *C*, cells stably expressing FLAG-LHR were transfected with APPL1-GFP and fed with AF555-labeled FLAG antibody (15min) prior to agonist stimulation (LH, 10 nm). Shown is a representative frame from live imaging of cells via confocal microscopy following 20 min of agonist stimulation. *Scale bar* = 5 μm.

To determine whether LHR endosomes represent a compartment that is either upstream or a distinct pathway from cargo trafficking to EEs, we employed fluorescently labeled transferrin to monitor the internalization of transferrin (Tf) and its receptor (TfR) following agonist-induced LHR internalization. Because we have shown previously that the LHR heterodimerizes with the B2AR (37), this precluded the coexpression of this receptor with the LHR. Tf/TfR, a classic marker of clathrin-mediated endocytosis and EEs, rapidly internalizes to a common EE pool, like the B2AR, prior to its sorting to the default plasma membrane recycling pathway (30). LHR-expressing cells were treated with labeled FLAG antibody prior to stimulation with LH for 15 min to induce receptor endocytosis. Cells were then treated with labeled Tf for the last 2, 5, or 10 min of LH treatment (Fig. 2, *A* and *B*). For cells treated with labeled Tf for 15 min, the labeled Tf was added simultaneously with LH (Fig. 2, *A* and *B*, *right column* and *right bar*, respectively). Therefore, for all Tf time points, all cells were stimulated with LH for 15 min only. Following 2 min of Tf treatment, many of the internalized labeled Tf colocalized with LHR-positive endosomes, with ∼43% of LHR endosomes positive for Tf (Fig. 2*B*). This colocalization decreased significantly following a 5 min treatment with labeled Tf, with only ∼10% of LHR endosomes positive for Tf. This is consistent with the rapid internalization of Tf to EEs within 5 min (38, 39). Because Tf transiently colocalizes with the LHR early on in the trafficking of Tf, this suggests that Tf rapidly internalizes through LHR endosomes.

The LHR internalizes to small endosomes, the majority of which do not colocalize with EE markers or EE-localized cargo, suggesting that this may represent a pre-EE compartment. The adaptor protein phosphotyrosine interaction, pleckstrin homology domain, and leucine zipper containing 1 (APPL1) has been demonstrated to be recruited to a pre-EE or intermediate compartment. Therefore, we asked whether the LHR traffics to APPL1-positive endosomes. We expressed APPL1-GFP in LHR-stable cells and treated them with labeled FLAG antibody prior to ligand stimulation. The LHR internalized into endosomes that were positive for APPL1-GFP when imaged via confocal microscopy, with 58.9 + 12.6% (*n* = 6) of LHR endosomes colocalizing with APPL1-GFP. Overall, these results demonstrate that the majority of the internalized LHR is targeted to a population of endosomes distinct from the EE, which may comprise a pre-EE stage in the postendocytic pathway.

##### Receptor Sorting to a Pre-EE Compartment Is Sequence-dependent

**FIGURE 3.**
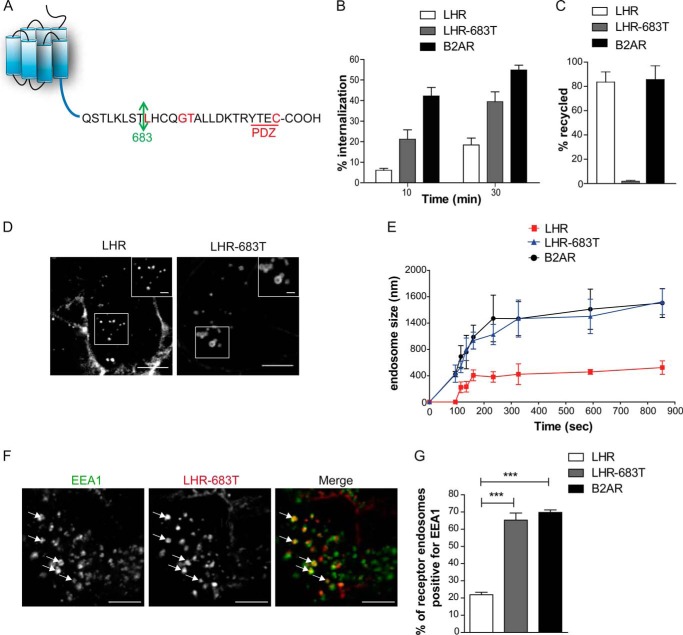
**Targeting of LHR to pre-EEs requires the distal C-terminal tail.** *A*, schematic of the LHR C-terminal tail. Residues highlighted in *red* have been described to be essential for receptor recycling (29, 40, 41). The *arrow* indicates the residue mutated to a stop codon to create truncation mutant LHR-683T. *B* and *C*, agonist-induced (LH, 10 nm; isoproterenol, 10 μm) internalization (*B*) and recycling (*C*) of the LHR, LHR-683T, and B2AR were quantitatively measured by flow cytometry. Data are mean ± S.E., *n* = 3. *D*, representative frame from a time-lapse movie of cells stably expressing either LHR or LHR-683T treated with an agonist, indicating the difference in size of endosomes to which each receptor internalizes. *Scale bars* = 5 μm and 1 μm (*insets*). *E*, average diameter of endosomes containing the LHR, LHR-683T, or B2AR following treatment with an agonist across the indicated times. For each time point, 10 endosomes are measured across three to four movies for each receptor. Data are mean ± S.E. *F*, representative confocal images of fixed cells stably expressing FLAG-LHR-683T following 30 min of agonist treatment and treated with anti-EEA1 antibody. The *arrows* indicate examples of colocalization of the receptor with EEA1. *Scale bars* = 5 μm. *G*, the percentage of receptor positive endosomes with EEA1 was quantified for the LHR, LHR 683T, and B2AR. Data are mean + S.E. (*n* = 15 cells, 220 endosomes for each receptor). ***, *p* < 0.001. See also supplemental Movie S3.

We next assessed whether the postendocytic sorting of the LHR to pre-EEs is a regulated, receptor-driven process. The distal region of the LHR C-terminal tail (C-tail) contains the sequence required for its plasma membrane recycling that also includes a known PDZ ligand (29, 40, 41) (Fig. 3*A*). The receptor was truncated to remove its recycling sequence and PDZ ligand (LHR-683T). Accordingly internalized LHR-683T is unable to recycle and thus exhibits greater internalization (Fig. 3*B*-C) as previously shown (40). However, upon live imaging of this LHR mutant, we observed that the receptor was able to internalize in to larger endosomes than the full-length LHR, with a similar size and kinetic profile to that of B2AR (Fig. 3*D*-E and Movie S3). Following 30 min of agonist treatment, a significantly greater number of LHR-683T endosomes were positive for EEA1, compared with LHR (Fig. 3*F*-G) indicating that the truncated receptor localized primarily to the EE compartment. These results demonstrate that the LHR distal C-tail is necessary for receptor targeting to small EEA1-negative endosomes.

##### GIPC Is Essential in Determining LHR Endosomal Targeting

**FIGURE 4.**
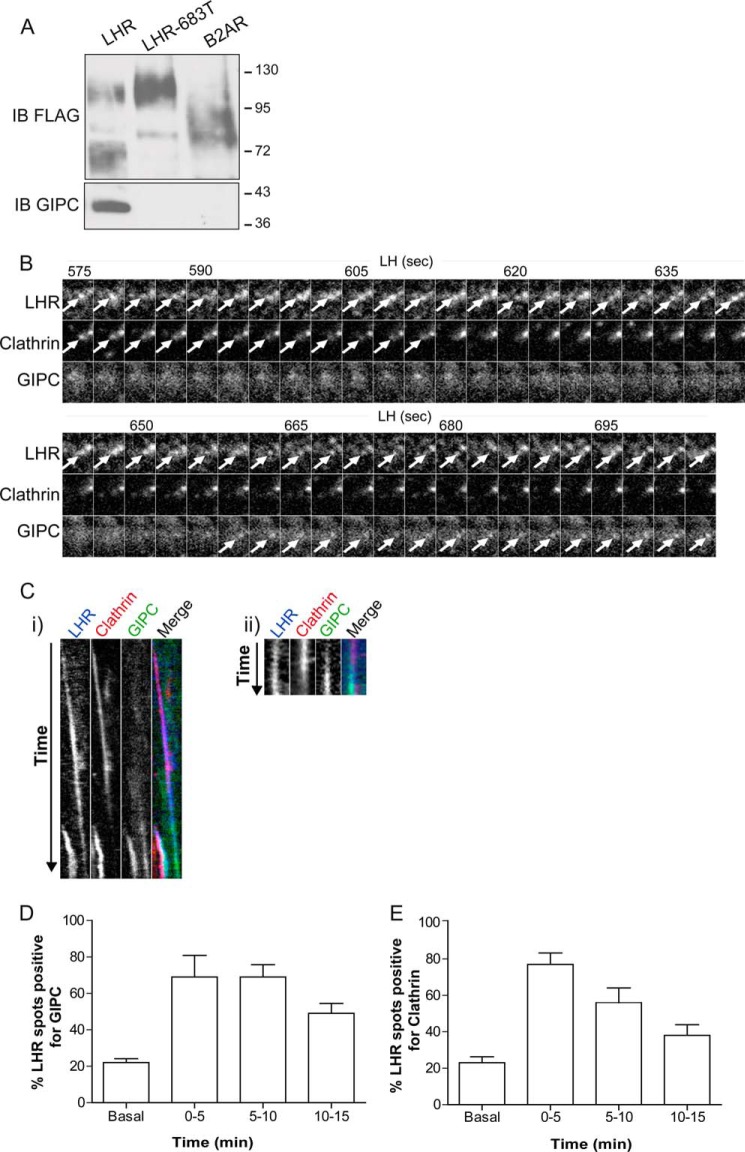
**The LHR associates with GIPC during agonist-induced internalization.** *A*, interaction of endogenous GIPC with FLAG-tagged receptors by coimmunoprecipitation, followed by immunoblotting (*IB*) of both FLAG and GIPC. *B*, total internal reflection fluorescence microscopy images from HeLa cells transiently transfected with FLAG-LHR, clathrin-DsRed, and GIPC-GFP. Representative time-lapse frames were taken every 3 s over a 900-s movie of LH treatment. Images shown are from 575–704 s following LH treatment to indicate LHR recruitment to clathrin followed by the appearance of GIPC when clathrin fluorescence decreases, indicating LHR internalization. The *arrows* indicate the appearance of LHR, clathrin, or GIPC fluorescence within the same spot. *C*, *i*, correspondent kymograph of the whole movie (0–900 s of LH treatment) shown in *B*. Note the appearance of a second colocalizing LHR/clathrin/GIPC spot, indicating that GIPC can be recruited with the appearance of the LHR in clathrin spots. *ii*, kymograph of a distinct movie taken every 3 s from 246–309 s following LH treatment, illustrating GIPC recruitment following the appearance of the LHR with clathrin. *D* and *E*, the total number of individual LHR spots colocalized with either GIPC (*D*) or clathrin (*E*) was counted from maximum projections at the indicated time points of LH stimulation. Data are mean ± S.E. across five independent experiments, representing a total of 209 spots for LHR/GIPC and 194 spots for LHR/clathrin.

Considering that the distal C-tail of the LHR was required for its endosomal localization, we next attempted to identify the mechanism underlying this pre-EE targeting. We started with known interacting proteins that bind to this region of the LHR C-tail. Both the B2AR and LHR interact with distinct PDZ domain-containing proteins with a high degree of specificity via PDZ ligands located in their distal C-tails (18, 40, 42). For the LHR, binding of the PDZ protein GIPC to the receptor C-tail has been shown to be required for recycling of its ligand (40). We confirmed, by coimmunoprecipitation, the requirement of the last 17 residues of the LHR C-tail for a GIPC interaction and that the B2AR does not interact with GIPC (Fig. 4*A*). Therefore, we first assessed how the LHR engages with GIPC on a spatial and temporal scale during ligand-mediated endocytosis. To monitor the early events of receptor trafficking, cells expressing the FLAG-tagged LHR, clathrin light chain-DsRed, and GIPC-GFP were imaged via total internal reflection fluorescence microscopy. Within 5 min of ligand stimulation, the LHR clustered into CCPs (Fig. 4, *B*, *C*, and *E*). GIPC was also recruited to the LHR within 5 min of ligand stimulation (Fig. 4, *B–D*). Tracking of GIPC and the LHR, in relation to CCPs/clathrin-coated vesicles (CCVs), revealed that GIPC was recruited early on in LHR trafficking, colocalizing with the LHR at the time of, or immediately after, the LHR localizes to clathrin spots (representing CCPs or CCVs) (Fig. 4, *Ci* and *Cii*, respectively). We also observed GIPC recruitment soon after loss of clathrin, indicating an association at the newly formed LHR endosome (Fig. 4, *B* and *Ci*). It also suggested that GIPC remains associated with the LHR after receptor internalization, indicated by the dissociation of clathrin (Fig. 4*C*, *ii*) and the temporal profiles of LHR localization with these two proteins (Fig. 4, *D* and *E*). These data demonstrate that GIPC is recruited to the LHR at an early stage in its trafficking.

**FIGURE 5.**
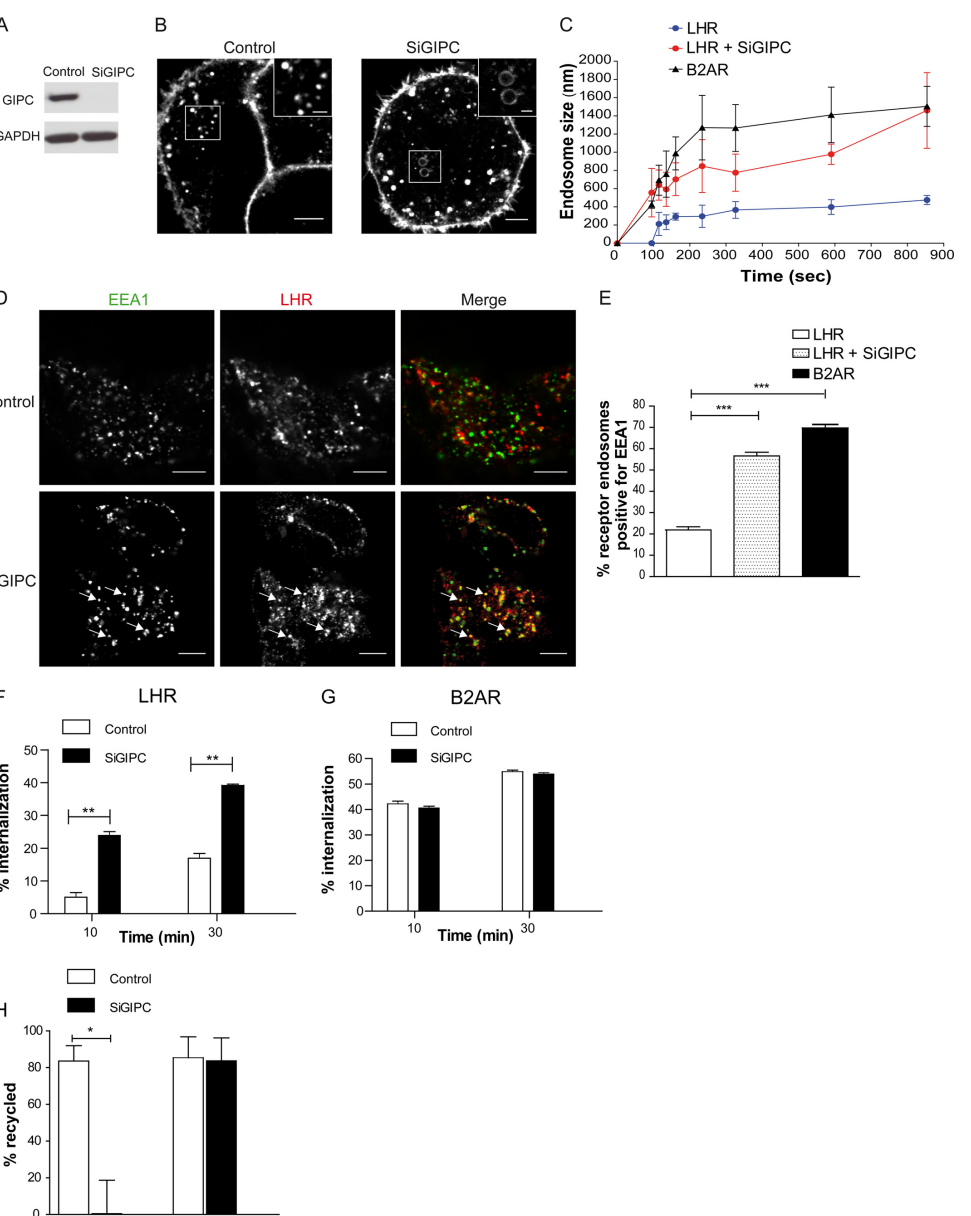
**Targeting of the LHR to pre-EEs requires GIPC.** *A*, representative Western blot analysis of total cellular levels of GIPC following siRNA-mediated knockdown as indicated. *B*, representative frames from live cell confocal imaging of LHR agonist-induced internalization following knockdown of GIPC (*SiGIPC*). *Scale bars* = 5 μm and 1 μm (*insets*). *C*, the size of LHR-containing endosomes was measured as in Fig. 1*B* in cells treated with either control or SiGIPC. The size of B2AR endosomes is also shown for comparison. Data are mean ± S.E. *D*, representative confocal images from fixed cells stably expressing FLAG-LHR following GIPC knockdown and stimulated with LH (10 nm) for 30 min, indicating that receptor internalized to endosomes that colocalize with EEA1 (*arrows*). *Scale bars* = 5 μm. *E*, the percentage of LHR-positive endosomes that visually colocalize with EEA1 in cells depleted of GIPC. Data are mean + S.E. (*n* = 28 cells, 450 endosomes) ***, *p* < 0.001. *F* and *G*, LHR and B2AR ligand-induced internalization and recycling following GIPC siRNA-mediated knockdown was analyzed by flow cytometry. Cells were treated with anti-FLAG antibodies to label the surface receptors prior to treatment with agonist (10 or 30 min with either LH (10 nm) or isoproterenol (10 μm)) to internalize the receptors. Cells treated for 30 min with the agonist were washed and incubated in medium for 1 h to allow for receptor recycling. The percentage of internalization refers to the fractional reduction of the surface receptor in response to agonist exposure for the LHR (*F*) and B2AR (*G*) **, *p* < 0.01. *H*, the percentage of receptor recycled refers to the fractional recovery of the surface receptor following agonist washout for 1 h. Data represent the mean ± S.E. from three independent experiments. *, *p* < 0.05.

To address whether GIPC is essential for the endosomal localization of the internalized LHR, we depleted cellular GIPC via siRNA. Live cell imaging of agonist-induced LHR internalization, in GIPC siRNA-depleted cells (Fig. 5*A*), exhibited a marked increase in the size of endosomes containing LHR compared with the control (Fig. 5*B*), with comparable diameters to the B2AR, within 15 min of agonist treatment (Fig. 5*C*). The increase in LHR endosome size was also accompanied by a significant increase in the number of receptor-containing endosomes positive for EEA1 (Fig. 5, *D* and *E*). The impact of routing the LHR to EEs on receptor postendocytic sorting fate following GIPC knockdown was assessed with flow cytometry. When surface receptor levels were quantitated with flow cytometry, GIPC-depleted cells exhibited a lower number of surface LHRs after agonist treatment (Fig. 5*F*) and strongly inhibited LHR recycling without impacting B2AR trafficking (Fig. 5, *G* and *H*), consistent with prior observations that GIPC knockdown inhibits the recycling of the ligand for the LHR (40). Overall, these data demonstrate an essential role for GIPC in targeting receptors to a pre-EE compartment, the loss of which reroutes their trafficking to EEA1-positive EEs. Further, it suggests that endosomal localization of the LHR to pre-EEs by GIPC drives postendocytic sorting of this receptor to a regulated recycling pathway.

##### Targeting of the LHR to Pre-EEs Is Essential in the Spatiotemporal Regulation of Signaling

**FIGURE 6.**
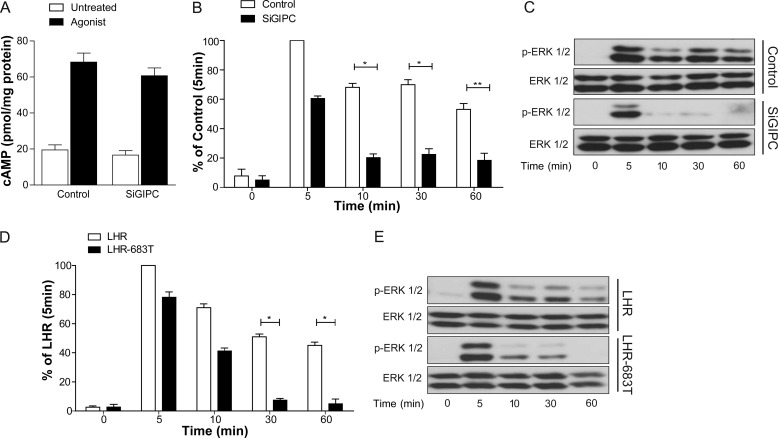
**Trafficking to pre-EEs is essential for a sustained LHR-mediated MAPK signaling profile.** *A*, intracellular levels of cAMP were measured in cells stably expressing the LHR following treatment with either control siRNA (*Control*) or GIPC siRNA (*SiGIPC*). Cells were stimulated with LH (10 nm) for 90 s. *B* and *C*, HEK 293 cells stably expressing LHR were treated with either control or GIPC siRNA, and phosphorylation of ERK 1/2 was determined by Western blotting. Total ERK was used as a loading control. For *B*, densitometric analysis of ERK 1/2 phosphorylation was normalized to the 5-min control stimulation. A representative immunoblot is shown in *C*. Data represent mean ± S.E. (*n* = 5). *D* and *E*, HEK 293 cells stably expressing either FLAG-LHR or FLAG-LHR-683T were treated with an agonist for the indicated times, and phosphorylation of ERK 1/2 was determined by Western blotting. For *D*, densitometric analysis of ERK 1/2 phosphorylation was normalized to the 5-min LHR stimulation. A representative immunoblot is shown in *E*. Data represent mean ± S.E. (*n* = 3). *, *p* < 0.05; **, *p* < 0.01.

Given the integral relationship that membrane trafficking has with cell signaling (7, 43), our findings that the LHR is primarily targeted to a pre-EE population for its postendocytic sorting led us to address whether the functional role of this compartment was to regulate receptor signaling at a spatial level. To test this, we used either LHR-683T or the knockdown of GIPC to alter the postendocytic localization of the LHR from EEA1-negative to EEA1-positive endosomes. The primary G protein signaling pathway activated by the LHR is Gαs, leading to increases in intracellular cAMP. In LHR-expressing cells, depletion of cellular GIPC, via siRNA, had no significant effect on the LH-dependent increase in intracellular cAMP levels (Fig. 6*A*). Therefore, we assessed downstream signaling responses of LHR activation to the MAPK pathway. LHR-mediated activation of MAPK signaling was assessed via the measurement of ERK 1/2 phosphorylation via Western blotting. The LHR activates ERK with a peak signal following 5 min of agonist stimulation with sustained levels of activated ERK following 60 min, albeit at a slightly reduced level (Fig. 6, *B–E*). In cells expressing either LHR-683T or the LHR under conditions of GIPC knockdown, the profile of ERK signaling was altered from a sustained to a transient response (Fig. 6, *B*–E). These signal responses were independent of the known role of GIPC in negatively regulating heterotrimeric Gαi G protein signaling (44) because treatment with pertussis toxin, an inhibitor of Gαi, had no effect on ligand-induced ERK phosphorylation by the LHR (data not shown). Consistent with the lack of effect on cAMP signaling, these results suggest a non-G protein requirement of GIPC in a sustained MAPK signaling response by the LHR and indicate a signaling requirement for directing receptors to pre-EEs.

**FIGURE 7.**
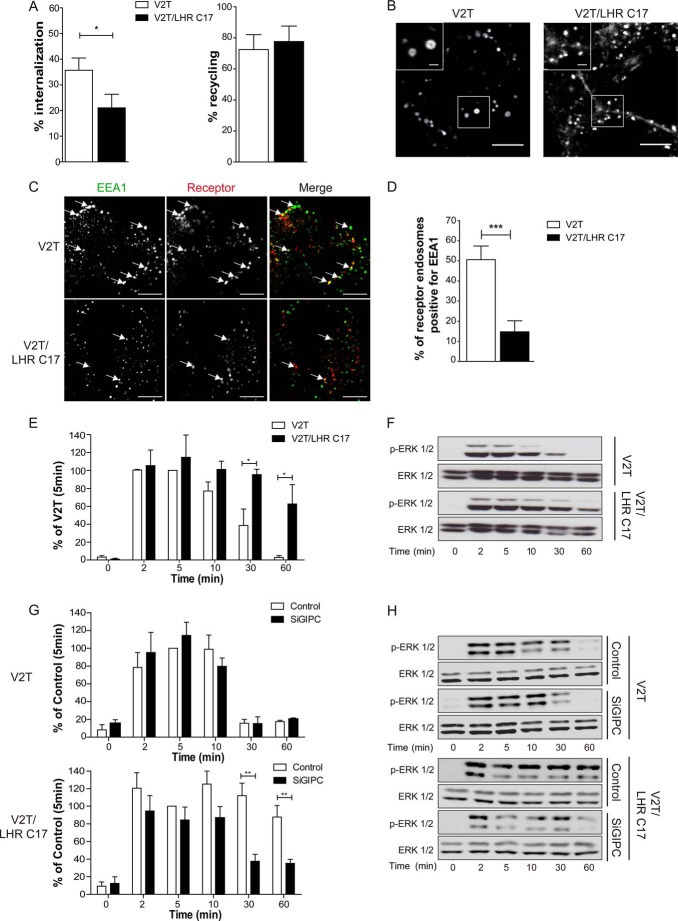
**The distal C-terminal tail of the LHR is sufficient to reroute EE-localized receptors to pre-EEs and a sustained MAPK signaling profile.** *A*, ligand-induced internalization and recycling of HEK 293 cells stably expressing FLAG-V2T or FLAG-V2T/LHR C17 was quantitatively measured via flow cytometry. Cells were treated with anti-FLAG antibodies to label the surface receptors prior to treatment with an agonist (30 min, arginine-vasopressin, 1 μm) to internalize the receptors. Cells were then washed and incubated in medium for 1 h to allow for receptor recycling. Surface receptor immunoreactivity was determined by flow cytometry. The percentage of internalization refers to the fractional reduction of the surface receptor in response to agonist exposure. The percentage of receptor recycled refers to the fractional recovery of the surface receptor following agonist washout for 1 h. Data represent mean ± S.E. from three independent experiments. *, *p* < 0.05. *B*, representative frames from live cell confocal imaging of cells expressing FLAG-V2T or FLAG-V2T/LHR C17 following agonist-induced internalization. *Scale bars* = 5 μm and 1 μm (*insets*). *C*, representative confocal images of fixed cells stably expressing FLAG-V2T or FLAG-V2T/LHR C17 following 30 min of agonist treatment (arginine-vasopressin, 1 μm) and treated with anti-EEA1 antibody. The *arrows* indicate examples of colocalization of the receptor with EEA1 *Scale bars* = 5 μm. *D*, the percentage of receptor-positive endosomes with EEA1 was quantified for V2R and V2T/LHR C17. Data are mean + S.E. (*n* = 10 cells, ∼280 endosomes for V2T, and 240 endosomes for V2T/LHR C17). ***, *p* < 0.001. *E* and *F*, HEK 293 cells stably expressing either FLAG-V2T or FLAG-V2T/LHR C17 were treated with arginine-vasopressin (1 μm) for the indicated time points. *E*, densitometric analysis of ERK 1/2 phosphorylation was normalized to the 5 min stimulation of V2T-expressing cells. Data represent mean ± S.E. (*n* = 3). *, *p* < 0.05. *G* and *H*, HEK 293 cells stably expressing either V2T or V2T/LHR C17 were treated with either control or GIPC siRNA (*SiGIPC*), and phosphorylation of ERK 1/2 was determined by Western blotting. Total ERK was used as a loading control. Representative immunoblots are shown in *H*. For *G*, densitometric analysis of ERK 1/2 phosphorylation was normalized to the 5-min control stimulation. Data represent mean ± S.E. (*n* = 4).

We then asked whether we could reroute a receptor normally localized in the EE to the pre-EE compartment and if so, whether it would conversely alter its signal response. To do this, we added the last 17 residues of the LHR to another rapidly recycling receptor, the vasopressin type 2 receptor lacking its C-tail (V2T), which we have utilized previously to study postendocytic sorting mechanisms of GPCRs (19, 22, 23, 45). This EE-localized receptor was used instead of the B2AR because the latter receptor requires multiple sequence domains across the C-tail for its sorting. Specifically, the B2AR requires three distinct sequences in its C-tail to undergo sorting via sequence-directed recycling. The distal PDZ ligand, (18), an upstream acidic dileucine-like sequence that determines dependence on the EE-localized protein Hrs/Vps27, (23), and thirdly, another upstream C-tail PKA site that regulates a “switch” between sequence-directed and default recycling (46). In contrast, V2T undergoes sorting via the default/bulk membrane pathway independently of Hrs/Vps27 and C-tail sequences (23). Addition of the last 17 residues of the LHR C-tail to V2T, to generate the chimera V2T/LHR C17, resulted in a receptor that underwent agonist-induced internalization, albeit less than V2T, but retained its ability to recycle (Fig. 7*A*). Live imaging of this chimera compared with V2T indicated that, although V2T internalized into large endosomes, V2T/LHR C17 trafficked primarily to small endosomes similar to the LHR (Fig. 7*B*). Furthermore, a significantly smaller number of V2/LHR C17 endosomes were positive for EEA1 compared with V2T (Fig. 7, *C* and *D*), indicating that V2/LHR C17 relocalized primarily to the EEA1 negative pre-EEs. The ERK signaling profile of the activated V2T was transient with a peak signal following 5 min of stimulation (Fig. 7, *E* and *F*). Interestingly, addition of the LHR C-tail to the V2T changed the pattern of ERK phosphorylation to a significantly more sustained signaling profile (Fig. 7, *E* and *F*). To confirm that the sustained ERK signaling response is due to a dependence on GIPC, cells expressing either V2T or V2/LHR C17 were treated with and without GIPC siRNA. Conditions of GIPC knockdown had no effect on V2T-mediated ERK signaling (Fig. 7, *G* and *H*). Similar findings were also obtained with B2AR-mediated ERK signaling (not shown). In contrast, the ERK signaling profile of V2/LHR C17 was altered from a sustained to a transient response (Fig. 7, *G* and *H*), as observed with the WT LHR (Fig. 6, *B* and *C*). Therefore, the LHR distal C-tail is both necessary and sufficient to control its endosomal localization and MAPK signaling profile.

**FIGURE 8.**
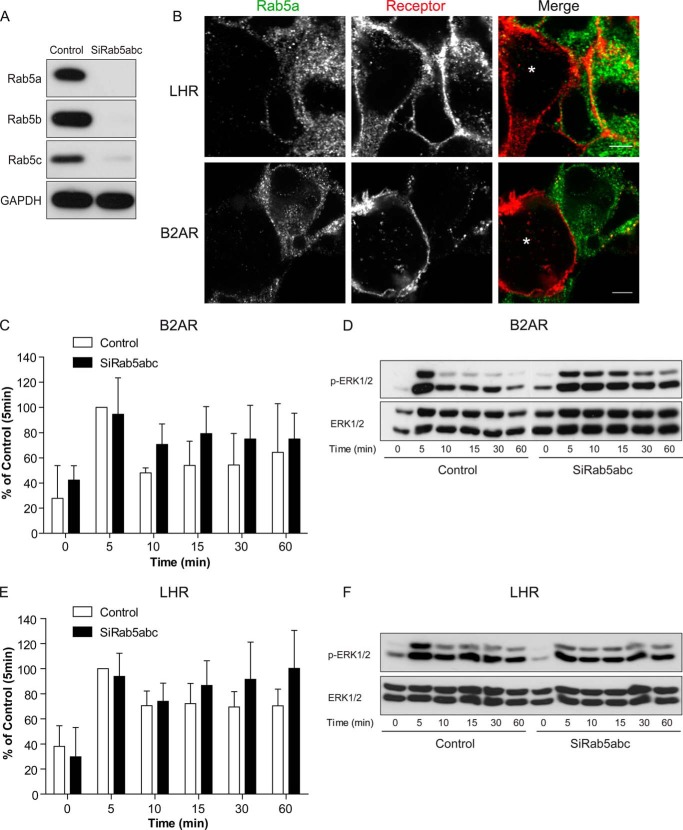
**LH-induced sustained ERK signaling is independent of Rab5.** *A*, representative Western blot analysis of total cellular levels of Rab5a, b, and c following siRNA-mediated knockdown of Rab5 a, b, and c. *B*, confocal images of cells expressing FLAG-B2AR or FLAG-LHR, treated with either control or Rab5 a/b/c siRNAs, were cocultured to directly compare Rab5-positive and -negative cells with receptor internalization within the same imaging field. Cells were fed with anti-FLAG antibody (*red*) and stimulated with ligand (10 nm LH or 10 μm isoproterenol) for 10 min for the B2AR or 30 min for the LHR before fixation, permeabilization, and treatment with anti-Rab5a antibodies (*green*). Cells effectively depleted for Rab5 are indicated by an *asterisk. Scale bars*, 5 μm. *C* and *D*, measurement of B2AR-mediated ERK 1/2 phosphorylation following Rab5 depletion. Cells were stimulated with isoproterenol (10 μm) for the indicated time points before lysis and immunoblotting. *C*, densitometric analysis of immunoblot analysis from three independent experiments. Data are normalized to 5-min stimulation of control siRNA-treated cells and represent mean + S.E. (*n* = 3). In *D*, a representative immunoblot analysis is shown with total ERK as a loading control. *E* and *F*, measurement of LHR-mediated ERK 1/2 phosphorylation following Rab5 depletion. Cells were stimulated with LH (10 nm) for the indicated time points before lysis and immunoblotting. *E*, densitometric analysis of immunoblot analyses from three independent experiments. Data are normalized to 5-min stimulation of control siRNA-treated cells and represent mean + S.E. (*n* = 3). In *F*, a representative immunoblot analysis is shown with total ERK as a loading control.

**FIGURE 9.**
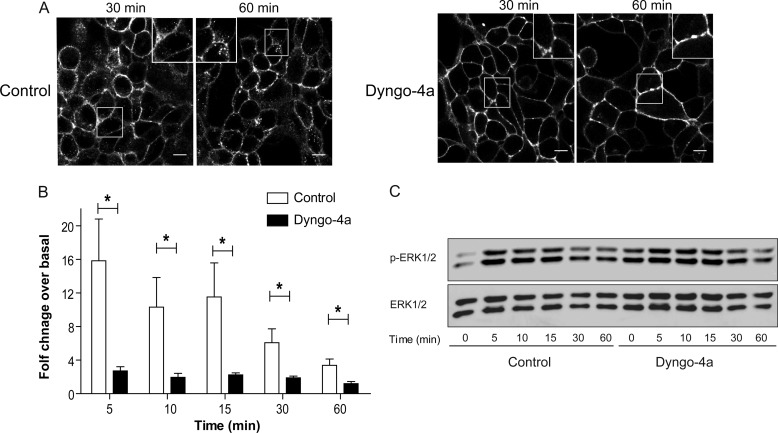
**Agonist-induced activation of ERK signaling by the LHR requires internalization.** *A*, agonist-induced internalization of the LHR was inhibited by pretreatment of cells with Dyngo-4a (30 μm) 15 min prior to LH stimulation (10 nm). Shown are representative frames from live confocal imaging of LHR-expressing cells following 30 or 60 min of agonist stimulation. *Scale bars*, 5 μm. *B* and *C*, LHR internalization was inhibited by pretreatment of cells with Dyngo-4a (30 μm) 15 min prior to LH stimulation (10 nm) for the times indicated, and ERK 1/2 phosphorylation was measured. In *B*, densitometric analysis of immunoblot analyses is expressed as agonist-induced fold change over basal and represents mean + S.E. (*n* = 5). *, *p* < 0.05. *C*, representative immunoblot analysis of LHR-mediated pERK 1/2 activation following LH (10 nm) stimulation for the indicated times. Total ERK 1/2 was used as a loading control.

To confirm that the LHR signaling profile was not dependent on receptor localization to an EE compartment, we knocked down a member of the Rab family of small GTPases, Rab5. Rab5 is another classic marker of EEs in addition to the Rab5 effector protein EEA1, regulating fusion of incoming endocytic vesicles with EEs as well as the homotypic fusion between EEs (10). Cellular depletion of all three Rab5 isoforms (a, b, and c) has been shown previously to disrupt the early endosomal-lysosomal system (47). Treatment of cells with siRNA to all three Rab5 isoforms depleted Rab5 to undetectable levels (Fig. 8*A*). Agonist-induced LHR internalization was still apparent following knockdown of Rab5a/b/c despite an effective decrease observed in internalization of the B2AR (Fig. 8*B*, the *asterisks* denote Rab5-depleted cells). In cells expressing the B2AR, loss of Rab5 resulted in a slightly more prolonged agonist-induced ERK signaling response (Fig. 8, *C* and *D*). That these increases were not significant may be due to the reduced, but not complete, inhibition of B2AR internalization observed in cells depleted of Rab5 (Fig. 8*B*). Importantly, Rab5 knockdown had no significant effect on agonist-induced LHR signaling to the MAPK pathway (Fig. 8, *E* and *F*). Therefore, to determine whether an endosomal localization of the LHR was required for its ERK activation, we utilized a known potent inhibitor of dynamin-mediated endocytosis, Dyngo-4a (48). Pretreatment of LHR-expressing cells with Dyngo-4a strongly inhibited agonist-induced receptor internalization, even following 60 min of LH stimulation (Fig. 9*A*). Measurement of ERK signaling under these conditions demonstrated an increase in basal pERK levels (Fig. 9*C*). This was also evident in cells stably expressing B2AR (data not shown) and suggests an increase in signal responses from distinct receptors in these cells that require internalization for signal termination (49). Likewise, agonist-induced ERK signaling by B2AR was prolonged in the presence of Dyngo-4a (data not shown). However, the LH-mediated ERK response was significantly inhibited across all time points in cells pre-treated with Dyngo-4a (Fig. 9*B*-C). Overall, these results suggest that LHR endosomal localization is required for MAPK signaling, independent of the Rab5/EE compartment.

##### Targeting of receptors to distinct endosomes may spatially control signaling for a subset of GPCRs

**FIGURE 10.**
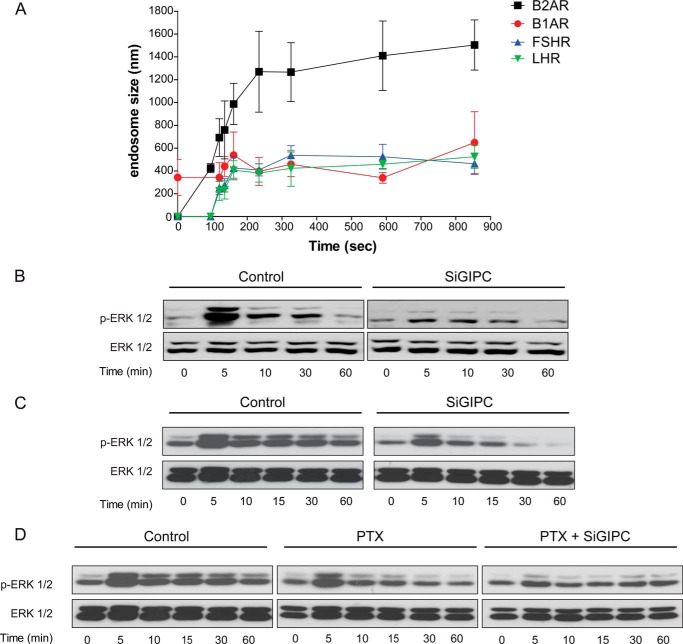
**Distinct GPCRs localized to small endosomes also require GIPC for a sustained MAPK signaling profile.** *A*, the size of FSHR- and B1AR-containing endosomes following agonist-induced internalization. Cells were treated with either FSH (10 nm) or isoproterenol (10 μm) following treatment with fluorescently labeled FLAG (FSHR) or HA (B1AR) antibodies. Endosome size was assessed by measuring the diameter of 10 endosomes at each time point stated across three movies. Data represent mean ± S.E. The data from Fig. 1*B* of LHR and B2AR endosome diameters are shown for comparison. *B* and *C*, representative immunoblot analyses of ligand-induced FSHR (*B*) and B1AR (*C*) ERK 1/2 phosphorylation treated with either control or GIPC siRNA (*SiGIPC*). Cells were treated with either FSH (10 nm) or isoproterenol (10 μm) for the indicated time points. *D*, cells expressing B1AR were pre-treated with pertussis toxin (*PTX*) (200 ng/ml, 18 h) with or without cotreatment of GIPC siRNA. Cells were then treated with isoproterenol (10 μm) for the indicated time points. Phosphorylation of ERK 1/2 was determined by Western blotting, and total ERK 1/2 was used as a loading control. A representative immunoblot analysis is shown.

We next asked if GPCRs in addition to LHR would require trafficking to distinct endosomal compartment to spatially regulate their MAPK signal responses. We analyzed the signaling profiles of two additional GPCRs we found to localize to small ‘LHR-like’ endosomes following agonist internalization; the follicle-stimulating hormone receptor (FSHR), and the beta1-adrenergic receptor (B1AR) (Fig. 10*A*). Ligand-induced ERK activation was measured in cells expressing either receptor following depletion of cellular GIPC. A requirement for GIPC in maintaining a sustained phospho-ERK profile was also observed for both the FSHR and B1AR (Fig. 10, *B* and C). Although GIPC has a known role in negatively regulating heterotrimeric Gαi G protein signaling of the dually Gαs and Gαi-coupled B1AR (44), cells that were pretreated with pertussis toxin only partially inhibited the activation of ERK 1/2 by the B1AR across the 60-min time course of agonist treatment, whereas GIPC knockdown in pertussis toxin treated cells further reduced the MAPK signaling response (Fig. 10*D*). Overall, these results suggest that the agonist-induced signaling response for a subset of GPCRs may be dictated by their targeted localization to distinct pre-EEs in a GIPC-dependent manner.

## DISCUSSION

Spatial control of GPCR signaling is an emerging concept that provides a mechanism for how cells translate complex signaling responses into defined cellular programs. Here we identify an unanticipated divergent organization in the endosomal trafficking of GPCRs sorted to the regulated recycling pathway and show that this receptor-driven endosomal targeting is critical for dictating specific signaling responses from these receptors.

**FIGURE 11.**
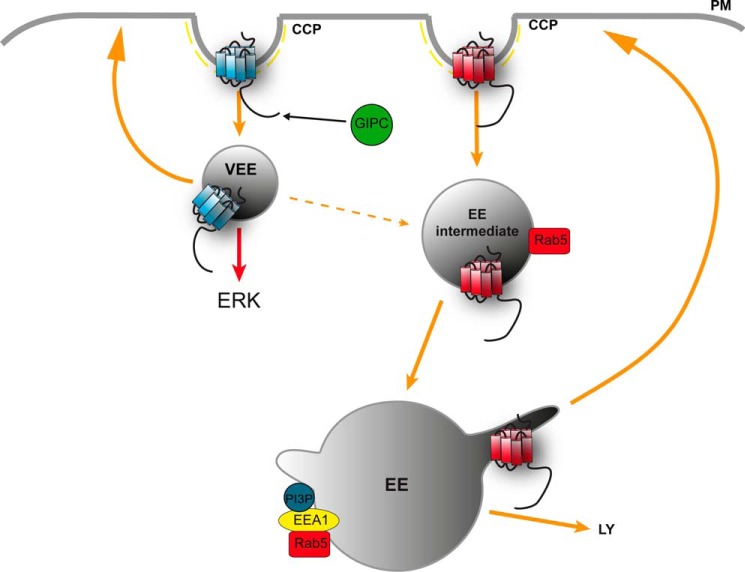
**Model depicting spatial restriction of GPCR signaling via divergent endocytic compartments.** Internalization of activated plasma membrane GPCRs via CCPs is targeted to pre-EEs or VEEs via recruitment and binding of the PDZ protein GIPC at the CCP. The VEE sorts receptors to the regulated plasma membrane recycling pathway, and targeting of GPCRs to this compartment generates a sustained MAPK signaling response. Receptors that do not bind GIPC are trafficked to the classic EE for subsequent sorting to the recycling pathway (regulated or default) or to the lysosome for degradation. The model depicts that VEEs and EEs may be interconnected compartments (*dotted arrow*), possibly via Rab5 endocytic intermediates.

The divergent postendocytic organization of two Gαs-coupled receptors, both of which are sorted to the regulated recycling pathway, into distinct endosomal compartments was first apparent by the striking difference in the size of LHR-containing endosomes compared with the B2ARs that classically traffic to EEs. Targeting receptors to these small endosomes was not a specific feature of the LHR because both the FSHR and B1AR also internalized to endosomes with a similar physical profile. The smaller size of these endosomes suggested that they were pre-EEs, consistent with prior observations of endosomal maturation properties (50, 51). Furthermore, that the majority of the LHR endosomes were both EEA1- and PI3P-negative is also consistent with LHR trafficking to a pre-EE compartment. Trafficking of the LHR to pre-EEs may serve as a very early postendocytic sorting platform for targeting this receptor to the regulated recycling pathway, whereas rerouting the LHR to the EE results in a loss of its recycling, despite the ability of other cargo to recycle from the EE via either bulk membrane (V2T) or sequence-dependent pathways (B2AR). This indicates that postendocytic sorting to the regulated recycling pathway can occur from two distinct types of endosomes. Although the EE is traditionally viewed as both the earliest and primary platform for receiving, organizing, and sorting cargo by multiple mechanisms, a subpopulation of Rab5 pre-EEs or EE intermediates consist of precursors of classic sorting EEs that recruit APPL1 (52, 53). It is possible that LHR trafficking to small pre-EEs may represent a further distinct population, observed recently with the EGF receptor (54), because LHR endosomes were positive for APPL1, yet its internalization and signaling were not sensitive to Rab5 depletion. These findings are also consistent with prior observations that a Rab5 dominant negative mutant can increase LHR recycling (55). We propose a model in which the LHR preferentially traffics to pre-EEs that may represent a very early endosome (VEE), either as a result of entering a distinct CCP-mediated endocytic pathway from EE-localized GPCRs or perhaps directly preceding known Rab5 endosomes (Fig. 11) as Tf transiently associates with LHR endosomes early on in its trafficking. Alternatively, the endocytic pathways that differentially target GPCRs to the EE or the pre-EE/VEE may be interconnected (Fig. 11), following neither a linear nor parallel pathway of cargo trafficking, a model consistent with the current knowledge of complex membrane trafficking networks (7, 56, 57).

Mechanistically, targeting of the LHR to pre-EEs requires an association of the receptor with the PDZ protein GIPC at CCPs, which consequently inhibits receptor traffic to EEs. GIPC has known prior roles in the regulation of Gαi signaling through interactions with GAIP/RGS19 and in trafficking of cargo to EEs (58–60). For the LHR, interaction with GIPC is essential for its targeting to the pre-EE compartment. Thus, we propose that such trafficking is the primary role for this GIPC-cargo interaction and a distinct function from roles reported previously of this protein in signaling and trafficking through the Rab5/EE endocytic pathway (58, 61–64). This highlights both the functionally distinct nature of this endosomal compartment and that diverse GPCR PDZ ligand/PDZ protein interactions may confer a high degree of functional specificity in addition to driving receptor sorting to the regulated recycling pathway. Indeed, many trafficking proteins, *e.g.* β-arrestin and Vps27/Hrs (1, 11, 65), play functionally diverse roles in membrane trafficking.

The endosomes to which LHR traffics potentially represent a novel platform for endosomal signaling. Endosomal signaling from GPCRs has been proposed primarily from studies illustrating the G protein-independent activation of signaling to pathways, such as the MAPK pathway, via β-arrestin scaffolds. As certain GPCRs cointernalize with β-arrestin, their signal activation could be mediated from endosomal compartments in addition to the plasma membrane. Furthermore, with recent studies reporting direct visualization of GPCR and G protein activity from endosomes, illustrating a direct spatial control of G protein signaling, the current view of GPCR signaling is changing (66–68). Our studies both support and add to this emerging view. The LHR-mediated sustained profile of ERK signaling requires both internalization and targeting to the correct endosomal compartment. This is underscored by our observations that the LHR distal C-tail is both necessary and sufficient for this sustained GIPC-dependent signaling profile. Furthermore, the ability of both V2T and V2T/LHR C17 to recycle, yet traffic to distinct endosomal compartments, suggests that it is the localization to a pre-EE or EE compartment, rather than the ability of a receptor to recycle, that determines the MAPK signaling profile. Thus, in addition to the compartmental bias in G protein signaling between the plasma membrane and EEs activated by a GPCR, there is compartmental bias in the cellular MAPK signaling response across distinct endosomes between GPCRs.

The transient ERK signaling response of the LHR in GIPC-depleted cells (or via truncation of the distal C-tail), as opposed to the more complete inhibition with Dyngo-4a treatment, may suggest that the early kinetics are G protein-mediated and that the sustained responses require GIPC and pre-EE targeting. As reported for other GPCRs, LHR-mediated cAMP production is partly dependent on receptor internalization (data not shown), because cAMP signaling is independent of GIPC. (This study may support this model.) Alternatively, the transient ERK signaling response by the LHR, upon loss of GIPC, may be due to transient trafficking through a pre-EE compartment while en route to the EE. In either model, we propose that the role of GIPC in ERK signaling is a consequence of its role in enriching receptors in the pre-EE or VEE (Fig. 11). However, whether GIPC could directly recruit and/or scaffold signaling proteins in this endosomal compartment to produce a sustained signaling profile has yet to be determined. Although this scaffolding function has been ascribed previously to β-arrestin, the sustained MAPK signaling profile of LHR is most likely not mediated by its interactions with β-arrestin because loss of GIPC does not alter receptor association with β-arrestins, and β-arrestin is only observed associating with the LHR at the plasma membrane and CCPs via total internal reflection fluorescence microscopy (data not shown), as shown previously for the B2AR (20). Overall, this work highlights the interconnected nature of membrane trafficking and cellular signaling in defining specific spatial and temporal patterns of signaling pathways commonly activated by GPCRs.

In conclusion, our study provides a novel view of how GPCR activity can be regulated at a spatial level. Furthermore, it provides a system that could enable the cell to reprogram its signaling for a diverse set of receptors by simply altering the endosomal localization of an individual GPCR, a model mechanistically consistent with a membrane network that must handle numerous specialized receptors with diverse cellular itineraries and functions.

## Footnotes

3 The abbreviations used are:

GPCR: G protein-coupled receptor
CCP: clathrin-coated pit
EE: early endosome
PDZ: postsynaptic density protein (PSD95), *Drosophila* disc large tumor suppressor (Dlg1) zonula occludens-1 protein (zo-1)
GIPC: GAIP-interacting protein C terminus
LH: luteinizing hormone
FSHR: FSH receptor
AF: Alexa Fluor
LHR: luteinizing hormone receptor
B2AR: β2-adrenergic receptor
PI3P: phosphatidylinositol 3-phosphate
Tf: transferrin
TfR: transferrin receptor
C-tail: C-terminal tail
VEE: very early endosome.
